# Developmental assets, creativity, thriving, and mental health among Malaysian emerging adults

**DOI:** 10.3389/fpsyg.2022.944238

**Published:** 2022-09-06

**Authors:** Nor Ba’yah Abdul Kadir, Helma Mohd Rusyda

**Affiliations:** Faculty of Social Sciences and Humanities, Center for Research in Psychology and Human Well-Being, Universiti Kebangsaan Malaysia, Bangi, Malaysia

**Keywords:** creativity, thriving, emerging adults, Malaysia, developmental assets, mental health

## Abstract

This study was part of a larger cross-national research project at the Norway’s University of Bergen, which involved participants from over 30 countries. This undertaking delves into developmental assets, creativity, and thriving, and the part they play in determining mental health. Thus, this study examined the developmental assets (internal assets: support, empowerment, boundaries, and expectations and creative use of time; external assets: commitment to learning, positive identity, positive values, and social competencies), creativity, thriving, and their importance to mental health in a sample of Malaysian emerging adults. This study was based on a sample of 394 undergraduate students, comprising 264 females and 130 males, ranging between the ages of 18 and 26 years (*M* = 21.5). Two subscales of the Reisman diagnostic creativity assessment (RDCA) were used to measure creativity (originality and fluency). Meanwhile, thriving indicators of Search Institute were used to measure thriving while the short form of the mental health continuum (MHC-SF) for adolescents was used to measure mental health. An online Google form was used to collect data from university students enrolled in both public and private universities. The correlation analysis results revealed that all the variables were positively correlated to each other and that the relationship between development assets, creativity, thriving, and mental health ranged from weak to moderate. Multiple regression (stepwise) analysis produced four models that indicated that positive identity, support, creativity, and thriving have a significant influence on mental health among emerging adults. Further, analyses using the PROCESS procedure demonstrated significant indirect effects of positive identity and support on mental health through its effects on creativity and thriving. As such, it is our recommendation that mental health practitioners tailor their treatment approach to include positive identity, support, creativity, and thriving in their sessions with Malaysian emerging adults.

## Introduction

A systematic literature review reported that the prevalence rate of mental health problems among university students in six Association of Southeast Asian Nations (ASEAN) countries (Cambodia, Laos, Malaysia, Myanmar, Thailand, and Vietnam) was 29.4% for depression, 42.4% for anxiety, 16.4% for stress, and 13.9% for disordered eating. Current suicidality was present in 7–8% of students (Dessauvagie et al., 2022). The high prevalence rate highlights the need to treat mental health issues through preventative measures and mental health promotion from an asset-based viewpoint, where resources and opportunities are provided. As such, research focusing on the importance of mental health, and its influencing factors, should be in the pipeline for the development of successful intervention programs. Hence, protective factors that might lead to mental health must be recognized to assist emerging adults in continuing to flourish and thrive in the face of frustration and despair.

The developmental assets framework and the positive youth development (PYD) framework have played a significant role in transforming the theoretical paradigm of youth development from deficit-based methods to asset-based methods. This is because the PYD offers youths environmental possibilities that may maximize their potential while deficit-based methods only prioritize addressing risks and problematic behaviors (Porter, 2010). Healthy youth are characterized in terms of positive attitudes and responsibilities that they are expected to attain in line with the strength-based approaches to youth development (Scales et al., 2000).

The present study was based on the developmental assets and PYD frameworks. The developmental assets framework is a vital resource with which to cultivate thriving (Search Institute, 1997). It consists of two categories: external assets, such as external structures, relationships, and activities; and internal assets, such as internal values, skills, and beliefs. Each category comprises four subcategories. External assets include (1) the support of family, school, and neighbors, (2) empowerment, (3) boundaries and expectations, and (4) the constructive use of time while internal assets include (1) commitment to learning, (2) positive identity, (3) positive values, and (4) social competence. The resources required to acquire developmental assets and skills can be found in a variety of sources, such as family, neighbors, colleagues, universities, and the religious community, to promote growth (Leffert et al., 1998; Benson et al., 1999), the capacity for change (Lerner, 2005), and for a better future (Adams et al., 2010). However, despite the importance of developmental assets among emerging adults, most studies have focused on developmental assets among children and adolescents only (Manrique-Millones et al., 2021). The most recent Malaysian studies focused on the correlation between developmental assets and character development (Arshad et al., 2021) and prosocial behaviors (Kaur et al., 2019) among youth. Meanwhile, other Malaysian studies examined at-risk youth (Abdul Kadir et al., 2012, 2014; Chong et al., 2012), disadvantaged youth (Ismail et al., 2015), Muslim youth (Krauss et al., 2014), and youth leaders (Arshad and Hong, 2019). Therefore, this present study was necessary to better understand the importance of developmental assets as a protective factor in the mental health of emerging adults.

The developmental assets have a positive correlation with mental health. Manrique-Millones et al. (2021) and Wiium et al. (2021) found that both internal and external assets significantly correlate with mental health. Previous studies found that all developmental assets negatively correlate with non-suicidal self-injury, particularly positive identity and empowerment (Taliaferro et al., 2020; Khan and Ungar, 2021; Zheng et al., 2021). This suggests that parents may more successfully promote protective factors for non-suicidal self-injury than friends. Furthermore, relationships with parents may have a protective effect against non-suicidal self-injury due to developmental assets, such as positive identity and empowerment (Taliaferro et al., 2020).

The World Health Organization (WHO) conceptualizes mental health as a “state of wellbeing in which the individual realizes his or her own abilities, can cope with the normal stresses of life, can work productively and fruitfully, and is able to make a contribution to his or her community” (World Health Organization, 2004). Keyes (2005) has outlined three components of mental health: emotional wellbeing, social wellbeing, and psychological wellbeing. Happiness, interest in life, and satisfaction are examples of emotional wellbeing; liking most of one’s own personality, being adept at handling daily obligations, having positive relationships with others, and being content with one’s own life are examples of psychological wellbeing; Positive functioning is referred to as social wellbeing, which includes having something to offer society (social contribution), feeling a part of a community (social integration), believing that society is improving for everyone (social actualization), and having a clear understanding of how society operates (social coherence).

Creativity contributes greatly toward good mental health (Tang et al., 2021; Zhai et al., 2021). The positive relationship between creativity and mental health, as revealed through previous studies (Blau and Benolol, 2016; Lin and Foley-Nicpon, 2019; Creech et al., 2020; Vilarinho-Pereira et al., 2021), is attributed to the flexible, independent, and self-accepting characteristics of human behavior (MacKinnon, 1965), as well as the positive influence of creativity, during bouts of loneliness (Michinov and Michinov, 2021). In this regard, Zhai et al. (2021) conducted a study on the association between emotional creativity and mental health, involving participants from different parts of China. According to the results attained, emotional creativity is negatively linked to specific mental health issues which include anxiety, depression, and somatization. Similarly, in an investigation on mental health, involving Turkish emerging adults, it was revealed that everyday creativity is negatively related to depression (Kircaburun et al., 2020). During the COVID-19 pandemic, creativity contributed toward the development of human endurance, strength, and resilience against the virus (Hölzle et al., 2020). According to Carson (2014), creativity can have a shielding effect against mental health issues, especially in emerging adults.

Creativity is defined in the context of product creativity, or a creative personality, which is also termed adaptive creativity (Simonton, 1997). This is an indication, that creativity necessitates the involvement of innovative, exceptional, unanticipated, and adjustable perceptions or products, which can deliver solutions to a variety of predicaments. In the opinion of Hayes (1989), creativity is an individual’s capacity for generating imaginative products, irrespective of whether he/she has done so before or never at all. Guilford (1950) equates creativity with uniqueness or innovation, and suitability or adjustability. Creativity is also described as a complicated phenomenon with several levels (Mumford et al., 2012), or in terms of a condition (Mumford and Gustafson, 1988; Runco and Jaeger, 2012). Everyday creativity, which is associated with imaginative solutions (Richards, 2007), is known as “Little c” creativity, while “Big C” creativity refers to cultural and historical transformations, associated with intellectual brilliance (Simonton, 2002).

In the opinion of Lerner et al. (2003), the thriving is achievable, if the individual: (a) contributes to society while improving his/her social life, (b) is supportive of social justice, (c) is dedicated to community activities, and (d) cherishes the closeness of family ties. The focus of previous investigations, in terms of the thriving among emerging adults, was mainly on its relationship with religious devotion (Buenconsejo, 2018), spirituality (Dowling et al., 2003; Gooden and McMahon, 2016), developmental assets (Scales et al., 2000; Desie, 2020), and the 5Cs of PYD (Tirrell et al., 2019). However, to date, investigations on thriving with regard to mental health, particularly among Malaysian emerging adults, is sorely lacking.

There are strong indications of a beneficial link, between thriving and mental health (Duan et al., 2020; Fuller-Thomson et al., 2020; Seppälä et al., 2020). Seppälä et al. (2020) investigated the association between psychological thriving and mental health by assessing the impact of three group interventions, on the mental health of university students. Burnout, stress, distress, anxiety, and depression were the mental health indicators, while psychological thriving condition indicators were psychological wellbeing, satisfaction, positive affect, negative affect, gratitude, self-compassion, mindfulness, maladaptive coping, optimism, self-esteem, and social connectedness. While the SKY Campus Happiness (SKY) intervention group was provided with stress-management training and the instruments for psychological resilience, the emphasis of the foundations in emotional intelligence (EI) intervention group was on knowledge of emotions as well as emotion regulation, and the mindfulness-based stress reduction (MBSR) intervention group was provided with training in three formal areas: mindfulness, meditation, and body scanning. In comparison to the control group and controlling for the variance of baseline measurements, as well as multiple comparisons, the SKY intervention group had the most profound effect, with gains associated with six outcomes, namely, depression, stress, mental health, mindfulness, positive affect, and social connectedness. Foundations in EI gained in one outcome, namely, mindfulness, while no changes were detected for the MBSR intervention group.

The thriving is described by Benson and Scales (2009), as a compilation of constructive “vital signs,” which include scholastic achievements, concern for others and their communities, acceptance of cultural and ethnic differences, and dedication to a healthy way of life. They opine that a thriving has to (a) depict a vibrant and two-way interaction of a young individual, who, over time, is invigorated at realizing his/her distinctive traits, and can identify those who recognize, support, applaud, foster, and promote their expressions, (b) entail steadiness or balance of movement toward an objective or the experience of equilibrium between developmental continuity and discontinuity over time, with regard to an individual’s relationships, and (c) indicate the current position of a young individual, on his/her route to idealized personhood, and whether the route taken can be considered ideally alterable.

There is a small body of literature that has focused on the relationship between developmental assets for positive identity, support, and mental health, which supports the potential indirect effects of developmental assets on mental health through creativity and thriving. Studies found that developmental assets were associated with thriving (Scales et al., 2000; Alvarado and Ricard, 2013) and creativity (Dimitrova et al., 2021). Studies also found that developmental assets for positive identity were related to higher creativity (Barbot and Heuser, 2017), and higher creativity was related to mental health (Acar et al., 2021; Attié et al., 2022). Overall, although these studies support the notion that creativity and thriving may be a mechanism of change in the relationship between developmental assets for positive identity and support for mental health, there is a need to test this mediational model. In addition, all of these studies were focused on adolescents. More research should examine these associations among emerging adults, especially about mental health. Although developmental assets are an important factor in the mental health of emerging adults (Garcia et al., 2019; Manrique-Millones et al., 2021; Wiium et al., 2021), the role that other factors, such as creativity and thriving, plays has not been thoroughly explored. This is especially true in the context of Malaysia.

## The present study

It is necessary to understand the associations between developmental assets, creativity, and thriving in mental health. In addition, no study has comprehensively explored the relations between the developmental assets, creativity, and thriving in the mental health of Malaysian emerging adults. Furthermore, there has been no in-depth investigation into how strongly each factor is related to mental health. Also, the association between the perception of developmental assets and mental health and the mechanisms through which developmental assets exert their influence on mental health have seldom been examined. Thus, this study is a first-of-its-kind investigation into the possible relations between developmental assets, creativity, and thriving on mental health among Malaysian emerging adults.

The following hypotheses and one research question were examined:

H_1_: Internal assets will be positively associated with mental health.

H_2_: External assets will be positively associated with mental health.

H_3_: Creativity will be positively associated with mental health.

H_4_: Thriving will be positively associated with mental health.

H_5_: Developmental assets (commitment to learning, positive values, social competencies, positive identity, support, empowerment, expectation and boundaries, and constructive use of time), creativity, and thriving, together will predict mental health.

H_6_: Developmental assets will have an indirect effect on mental health through their effect on creativity.

H_7_: Developmental assets will have an indirect effect on mental health through their effect on thriving.

## Materials and methods

### Settings and participants

This present study involved the participation of 394 students, from 15 Malaysian universities, with each university providing at least 20 participants. A total of 13 universities were located in the Peninsular Malaysia, while two universities were located on the island of Borneo (Sabah and Sarawak). The inclusion criteria of this present study was emerging adults aged 18 and 26 years who were formally registered as undergraduate students and Malaysian citizens. Emerging adults were excluded from this present study if they were unable to read or write or if they had been hospitalized for mental health issues within 6 months before the commencement of the study.

The ages of the participants ranged between 18 and 26 years (*M* = 21.51, *SD* = 1.21). Females make up 67% (*n* = 264) of the 394 participants and males 33% (*n* = 130). In the context of religious affiliation, 80.5% (*n* = 317) are Muslims, 7.1% (*n* = 28) are Hindus, 6.3% (*n* = 25) are Buddhists, 3.0% (*n* = 12) are Evangelic, 1.0% (*n* = 4) are Catholics, 0.5% (*n* = 2) are devotees of Kong Hu Chu, and 0.3% (*n* = 1) believes in mysticism. The majority of participants (89.1%, *n* = 351) are fully committed to their respective religious beliefs. In terms of their dwelling situation, 75.9% (*n* = 299) of the participants reside with their parents, 8.6% (*n* = 34) reside solely with their mother, 1.5% (*n* = 6) reside solely with their father, 6.1% (*n* = 24) with adults who are not their parents, 4.8% (*n* = 19) reside on their own, and 3.0% (*n* = 12) as much with mother as with father. As for their academic performance, 13.7% (*n* = 54) graded theirs as excellent, 30.2% (*n* = 119) as very good, 44.7% (*n* = 44.6) as good, 10.9% (*n* = 43) as fair, and 0.5% (*n* = 2) as poor.

This research was conducted as part of a larger cross-national research project at the University of Bergen in Norway. The larger research is about PYD and encompasses more than 30 nations. Thus, ethics approval was sought from the Institutional Review Board (IRB) of the University of Bergen (reference number 612969), with its separate study procedures outlining data aggregation across locations for analysis and dissemination. It was carried out in conformity with the Helsinki Declaration’s criteria and Malaysian Guideline for Good Clinical Practice.

The participating undergraduate students were given an online version of this survey. The institutions chosen were those that were most conveniently accessible to the study team, hence the student participants were drawn from a convenience sample. The cooperation of academics, based at the universities with survey-participating students, was secured for assistance during the study and for the allocation of the online survey. Student associations were roped in to spread the online link and encourage the response from students to the survey questions. Before the commencement of the online survey, the participants were provided with a concise explanation of the motives and goals of the study. Each participant required between 40 and 45 min to complete the online Google Form survey. As the participants in this data gathering process are willing volunteers, no refusal rate calculation was necessary. Also, due to the cross-sectional structure of this study, only a single data collection process is used.

### Measures

The translation of the questionnaire, from English to Malay, was carried out by two multilingual interpreters, who conformed to the cross-cultural translation benchmark requirements, as proposed by Beaton et al. (2000). Between the translators, while one is knowledgeable about the concepts of developmental assets, creativity, thriving, and mental health, the other is in the dark, regarding these concepts and themes related to psychology. The translators and researchers deliberated on differences in the items, and adjustments were made to the questionable wording of any item. A pilot test, involving 65 undergraduate students (age 21–26 years) was performed, to ascertain whether the items are corresponding. The values of Cronbach’s alpha for developmental assets (α = 0.95), creativity (α = 0.91), and mental health (α = 0.95) were acceptable, meanwhile Cronbach’s alpha for thriving was low (α = 0.43). Cohen (1988) and Hemphill (2003) recommendations were brought into play to ascertain the values of correlation coefficient (0.90–1.00 extremely high, 0.70–0.90 high, 0.50–0.70 moderate, 0.30–0.50 low, 0.01–0.30 weak). Subsequently, the final Malay edition, of the questionnaire was presented for the survey.

#### Demographic questions

Participants were required to provide demographic information which included their gender and age, and a self-evaluation report on their academic performance. The response to “How would you rate your academic performance?” is in accordance with a 5-point Likert scale, ranging from poor to excellent.

#### Developmental assets

The Search Institute’s Developmental Assets Profile was utilized (Scales, 2011). It consists of 58 items and has two main dimensions with four subscales on each (four internal assets and four external assets).

The external assets dimension is divided into four subscales: Support (7 items), empowerment (6 items), boundaries and expectations (9 items), and constructive use of time (4 items). The internal assets dimension is divided into four subscales: Commitment to learning (7 items), positive values (11 items), social competencies (8 items), and positive identity (6 items). Items are scored on a 4-point Likert scale ranging from not at all or rarely (1) to extremely or almost always (4). Summative scores ranged from 26 to 104 for external assets, with a higher total score denoting greater external assets, and 32–128 for internal assets, with a higher total score denoting greater internal assets. Previous studies demonstrated that this scale has good factorial and convergent validity in a sample of adolescents and youth (Scales, 2011). The values of Cronbach’s alpha were adequate for all subscales in this study (see Table 1), while a greater score on the developmental assets denotes a greater degree of developmental assets.

**TABLE 1:** Reliability test for developmental assets, creativity, thriving, and mental health.

Variables	Cronbach’s alpha	Number of items
Internal assets (total)	0.95	32
Commitment to learning	0.87	7
Positive values	0.87	11
Social competencies	0.85	8
Positive identity	0.85	6
External assets (total)	0.92	26
Support	0.80	7
Empowerment	0.79	6
Expectations and boundaries	0.84	9
Constructive use of time	0.64	4
Creativity	0.96	9
Thriving	0.79	8
Mental health	0.96	14

#### Creativity

The awareness of the participant, with regard to his/her creativity, was measured with the use of two subscales of the Reisman Diagnostic Creativity Assessment (RDCA, Reisman et al., 2016). Originality was gauged through six items, while fluency was gauged by way of three items. The choices for a response on the 5-point Likert scale are: strongly agree, agree, neither agree nor disagree, disagree, and strongly disagree. “I can come up with novel uses for things,” “I come up with new and unusual ideas,” and “I am innovative” are examples of items associated with originality, while “I can generate many solutions,” “I can produce a lot of ideas,” and “I generate many ideas” are examples of items related to fluency. Summative scores ranged from 6 to 45 for creativity with a higher total score denoting greater creativity. For this undertaking, Cronbach’s alpha value for creativity is deemed acceptable (α = 0.96), while a greater score on the RDCA subscale denotes a greater degree of creativity.

#### Thriving

The thriving was measured using the eight thriving indicators, developed by Scales et al. (2000) with the Search Institute. A “yes” or “no” answer was required from participants, in response to six items on a binary type scale. An example of items regarding the thriving include: “I engage in physical activity (for a minimum of 30 min) two or more times every week,” “I pay attention to healthy nutrition and exercise,” and “I have been a leader of a group or organization in the last 12 months.” One item measured helping others (e.g., How many hours do you spend in a typical week to help friends or neighbors?), one item on university success which was measured based on student self-report of grades, and one item on valuing diversity. For data analysis, a composite score, reflecting the number of thriving was created from the eight items, with scores ranging from 0, where the individual did not experience thriving at all, to 21, where the individual experiences greater thriving. For this study, the Cronbach’s alpha value, with regard to the thriving, is rated acceptable (α = 0.79), while a greater total thriving score denotes a greater thriving degree.

#### Mental health

The frequency of a positive mental health episode occurrence, within the past month, was determined by way of the Adolescent Mental Health Continuum-Short Form (MHC-SF) (Keyes, 2002, 2009). The initial 14-item MHC-SF (Keyes, 2005) comprises three items for computing emotional wellbeing, five items for computing social wellbeing, and six items for computing psychological wellbeing. While emotional wellbeing alludes to positive sentiments, social wellbeing alludes to social contribution, social integration, social actualization, social acceptance, and social coherence. Psychological wellbeing alludes to self-acceptance, environmental mastery, positive relations with others, personal growth, autonomy, and purpose in life. The participants’ response to items on a 6-point Likert scale is based on their experiences, for 1 month. The response choices are: “never,” “once or twice,” “about once a week,” “twice or thrice a week,” “almost every day,” and “every day.” Summative scores ranged from 14 to 84 for mental health with a higher total score denoting greater mental health. For this present study, Cronbach’s alpha value is deemed acceptable (α = 0.96), while the MHC-SF total score was computed as an indicator of complete mental health. As such, a greater score denotes a greater degree of mental health.

### Data analysis

The distribution and descriptive analysis statistics were obtained through the Statistical Package for the Social Sciences (SPSS) version 28. The data were screened for outliers and assumptions for parametric tests. Pearson’s product-moment correlation coefficients were used to explore the associations between variables. Then, multiple regression (Stepwise) analysis was performed to determine the factors that affect mental health. Before the execution of multiple regression analysis, basic assumptions such as normal distribution, skewness, kurtosis, and outliers were examined. As shown in Table 2, the minimum requirement for the skewness and kurtosis values were also met. The value obtained for skewness indicates a symmetric distribution for all the variables, while kurtosis implies a distribution that is neither too peaked nor too flat.

**TABLE 2:** Descriptive statistics of the study variables.

Variables	Minimum score	Maximum score	Mean	Standard deviation	Skewness	Kurtosis
Commitment to learning	1	4	3.19	0.52	0.50	0.50
Positive values	1	4	3.16	0.48	–0.35	0.42
Social competencies	1	4	3.19	0.51	–0.57	0.81
Positive identity	1	4	3.00	0.58	–0.46	0.29
Support	1	4	3.10	0.53	–0.53	0.27
Empowerment	1	4	3.06	0.54	–0.29	0.14
Expectations and boundaries	1	1	3.02	0.53	–0.40	0.54
Constructive use of time	1	4	2.61	0.66	–0.07	–0.34
Creativity	6	45	3.51	0.80	–0.42	0.21
Thriving	3	21	20.42	2.60	0.09	0.25
Mental health	14	84	4.47	1.02	–0.55	–0.44

**FIGURE 1:**
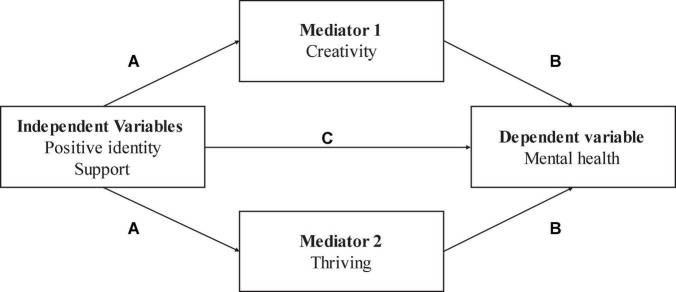
Hypothesized relationships among subscales of developmental assets, creativity, thriving, and mental health.

We conducted a mediation analysis to test the proposed indirect effects model suggesting that the association between developmental assets and mental health may be due, at least in part, to creativity and thriving. The PROCESS SPSS-28 macro v4.0 was used to analyze mediating effects depicted in Figure 1 (Preacher and Hayes, 2004). A sampling distribution was generated, with a 95% confidence interval, to test for the mediating effect. This study used bootstrap confidence intervals (CIs) to determine the significance of the effects based on 10,000 random samples. If the CIs do not include zero, then the effect is significant (Preacher and Hayes, 2008).

## Results

### Associations between developmental assets, creativity, thriving, and mental health

The application of the outlier labeling rule verified the non-existence of outliers (Hoaglin and Iglewicz, 1987). The calculation of bivariate Pearson’s correlations sheds light on the relationship between developmental assets, creativity, thriving, and mental health (Table 3). All the variables, in this investigation, are markedly interrelated in the anticipated paths. Computations, in compliance with the broad guidelines presented by Hair et al. (2014), revealed weak to moderate interrelations among all the variables. With regard to hypothesis 1, a significant positive link was showed between internal assets and mental health (*r* = 0.52, *p* < 0.01). A significant positive link was also showed for hypothesis 2, between the external asset and mental health (*r* = 0.45, *p* < 0.01). A significantly positive link was also perceived for hypothesis 3, between creativity and mental health (*r* = 0.47, *p* < 0.01), and for hypothesis 4, thriving and mental health (*r* = 0.22, *p* < 0.01). These positive coefficients serve to reveal that the rise in the value of one variable triggers the rise in the value of all the other variables. The inter-correlation of the variables under investigation verifies the non-occurrence of overlapping. Thus, the correlation coefficient between developmental assets, creativity, and thriving in mental health ranged from weak to moderate. No correlation was found between empowerment, boundaries and expectation, and constructive use of time on thriving.

**TABLE 3:** Correlation analysis between developmental assets, creativity, thriving, and mental health.

Variables	1	2	3	4	5	6	7	8	9	10	11
Commitment to learning	−										
Positive values	0.75**	−									
Social competencies	0.68**	0.77**	−								
Positive identity	0.64**	0.64**	0.69**	−							
Support	0.54**	0.53**	0.54**	0.53**	−						
Empowerment	0.58**	0.58**	0.57**	0.58**	0.69**	−					
Expectations and boundaries	0.57**	0.55**	0.57**	0.53**	0.73**	0.73**	−				
Constructive use of time	0.41**	0.40**	0.38**	0.48**	0.50**	0.54**	0.55**	−			
Creativity	0.33**	0.33**	0.34**	0.47**	0.30**	0.33**	0.24**	0.36**	−		
Thriving	0.20**	0.17**	0.17**	0.14**	0.14**	0.08	0.08	0.06	0.17**	−	
Mental health	0.41**	0.43**	0.47**	0.53**	0.40**	0.41**	0.36**	0.33**	0.47**	0.22**	−

**p < 0.01. All tests are two-tailed.

### Examine the influences of developmental assets, creativity, and thriving on mental health

Stepwise multiple regression analysis was performed to determine the factors predicting mental health. The variables (age and gender) and the subscales of developmental assets, creativity, and thriving, were included in the model. Gender and age were regarded as covariates. Dummy variables were conceived for gender. The rummaging mode response to this hypothesis 5 led to the stepwise removal of variables with non-significant effects. The entry of gender and age preceded the entry of the subscales of developmental assets, creativity, and thriving.

Model 1 showed that positive identity explained 29% of the variance in mental health (*R*^2^ = 0.29; *F*_change_ = 163.66; *p* < 0.001). In other words, the strongest predictor of mental health was found to be a positive identity. A positive beta value indicates that the mental health score increases as the positive identity score increases (beta = 0.54). Thus, positive identity was found to positively affect mental health (Table 4).

**TABLE 4:** Stepwise multiple linear regression models regarding the prediction of mental health (*n* = 394).

Model		Unstandardized coefficients		Standardized coefficients	*t*	Sig.	Collinearity statistics	
					
		*B*	*SE*	Beta			Tolerance	Variance Inflation Factor
1	Positive identity	0.95	0.07	0.54	12.79	< 0.001	1.000	1.000
2	Positive identity	0.73	0.08	0.41	8.98	< 0.001	0.777	1.287
	Creativity	0.34	0.06	0.27	5.87	< 0.001	0.777	1.287
3	Positive identity	0.72	0.08	0.34	6.65	< 0.001	0.613	1.631
	Creativity	0.33	0.06	0.26	5.72	< 0.001	0.774	1.292
	Support	0.28	0.02	0.15	3.07	0.002	0.718	1.392
4	Positive identity	0.60	0.09	0.34	6.64	< 0.001	0.613	1.632
	Creativity	0.31	0.06	0.25	5.38	< 0.001	0.762	1.312
	Support	0.26	0.09	0.14	2.88	0.004	0.714	1.400
	Thriving	0.04	0.02	0.12	2.84	0.005	0.961	1.041

		***R* ^2^**	**Adjusted *R*^2^**	***R*^2^ change**	***F***	***F-*change**	***P***	**Durbin–Watson**

	Model 1	0.29	0.29	0.29	163.66	163.66	< 0.001	1.89
	Model 2	0.35	0.35	0.06	106.09	34.52	< 0.001	
	Model 3	0.37	0.36	0.02	75.38	9.41	0.002	
	Model 4	0.38	0.37	0.01	59.51	8.05	0.005	

In model 2, the test was performed after adding creativity to the model along with positive identity. With this addition, the explained variance in mental health increased from 29 to 35% (*R*^2^ = 0.35; *F*_change_ = 34.52; *p* < 0.001). The creativity made a contribution of about 6% to the explained variance. A positive relationship was found between creativity and mental health (beta = 0.27), thus, as the creativity score increased, the mental health score increased. Thus, creativity was found to positively affect mental health (Table 4).

In model 3, the support variable was added along with positive identity and creativity, and with this addition, the explained variance in mental health score increased from 35 to 37% (*R*^2^ = 0.37; *F*_change_ = 9.41; *p* < 0.001). The support made a contribution of about 2% to the explained variance. A positive relationship was found between support and mental health (beta = 0.15), as the support score increased, the mental health score increased. Thus, support was found to positively affect mental health (Table 4).

In Model 4, the thriving variable was added along with positive identity, creativity, and support, and with this addition, the explained variance in mental health score increased from 36 to 37% (*R*^2^ = 0.38; *F*_change_ = 8.05; *p* < 0.001). The thriving made a contribution of about 1% to the explained variance. A positive relationship was found between thriving and mental health (beta = 0.12), as the thriving score increased, the mental health score increased. Thus, thriving was found to positively affect mental health (Table 4).

The *t*-test results regarding the beta coefficients of the variables included in the model at the fourth step and the significance of these coefficients that the positive identity (beta = 0.34), creativity (beta = 0.25), support (beta = 0.14), and thriving (beta = 0.12) variables significantly predicted mental health. Considering the beta values of the variables in the model, the Malaysian emerging adults’ mental health was significantly predicted by positive identity, followed by creativity, support, and thriving, respectively.

Other subscales of the developmental assets such as commitment to learning, positive values, social competencies, empowerment, expectation and boundaries, and constructive use of time were not included in the regression model since they did not have a significant effect on predicting the mental health.

### Test of indirect associations

**FIGURE 2:**
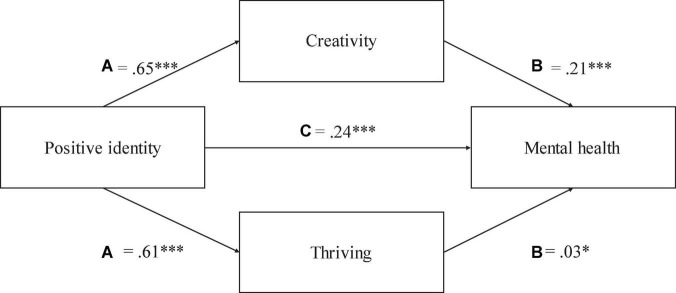
The regression coefficients for path **(A,B)** were significant, representing effects of positive identity on creativity and thriving, and effects of creativity and thriving on mental health. The regression coefficient for path C was also significant. * meaning significance level as follow: *p < 0.05, ***p < 0.001.

**FIGURE 3:**
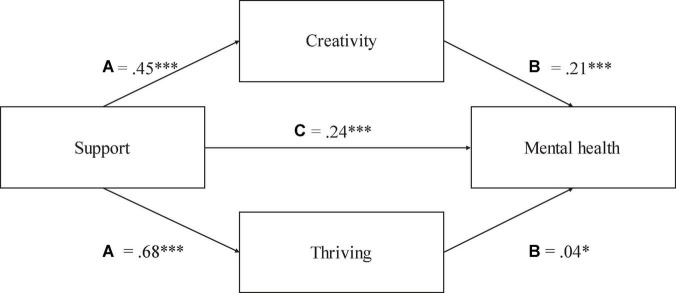
The regression coefficients for path **(A,B)** were significant, representing effects of support on creativity and thriving, and effects of creativity and thriving on mental health. The regression coefficient for path C was also significant. * meaning significance level as follow: *p < 0.05, ***p < 0.001.

To test hypotheses 6 and 7, two sets of analyses were conducted using model number 4 in the PROCESS SPSS macro v4 (Preacher and Hayes, 2004) to test for the possibility of indirect effects regarding creativity and thriving as indirectly associated with positive identity and support in mental health. The direct effect of positive identity on mental health was significant [*b*_simple_ = 0.71, *t* = 8.87, *p* < 0.001, CIs (0.5552, 0.8715)]. The results also showed that positive identity was indirectly associated with mental health through its association with creativity and thriving. It can be seen in Figure 2 that the indirect effect of positive identity on mental health through creativity [*b*_simple_ = 0.21, CI (0.1128, 0.3206)] and thriving [*b*_simple_ = 0.03, CI (0.0039, 0.0656)] was significant. Hence, creativity and thriving appeared to have significant indirect associations with positive identity and mental health. The possibility of indirect effects regarding creativity and thriving in relation to support and mental health was also tested. It can be seen in Figure 3 that the direct effect of support on mental health was significant [*b*_simple_ = 0.54, *t* = 6.31, *p* < 0.001, CIs (0.3706, 0.7063)]. The indirect effect of support on mental health *via* creativity [*b*_simple_ = 0.21, CI (0.1211, 0.3128)] and thriving [*b*_simple_ = 0.04, CI (0.0042, 0.0739)] was also significant. Hence, creativity and thriving appeared to have significant indirect associations with support and mental health.

## Discussion and conclusion

This study examined the relationship between developmental assets, creativity, thriving, and mental health in a sample of Malaysian emerging adults. Results showed that all variables were significantly positively correlated with mental health. This study also examined the indirect association of creativity and thriving with mental health among Malaysian emerging adults. The results indicated that positive identity significantly correlates with mental health and that creativity and thriving mediate this correlation. Similarly, support was found to significantly correlate mental health with creativity and thriving mediating this correlation as well. Similar to the findings of extant studies, this present study found that positive identity positively correlates with mental health (Wu and Ou, 2022) and negatively correlates with depression (Zhou et al., 2020). Other studies also found that support significantly correlates with complete mental health (Xu et al., 2019) and negative correlates with mental health issues (Hou et al., 2020). Therefore, these findings confirm the importance of positive identity and support as predictors of mental health.

The findings of other studies indicate that support significantly correlates with mental health and that creativity and thriving mediate the correlation. The findings of this present study are also corroborated by that of previous studies that report a positive correlation between support and the mental health of emerging adults as well as the mediating role of creativity and thriving. Similar to previous studies, emerging adults who received support from family and others have a positive correlation with mental health (Qi et al., 2020). This suggests that efforts to improve the mental health of emerging adults should focus on enhancing support from parents, peers, and faculty.

In this study, Malaysian emerging adults were examined in terms of the relationships between their developmental assets, creativity, thriving, and mental health. In keeping with the results from all hypotheses, all variables are significantly, and positively, interrelated with complete mental health. The findings derived through this study are in agreement with those from earlier investigations (Paek et al., 2016; Michinov and Michinov, 2021). Apparently, these findings indicate that the rise in the degree of developmental assets, creativity, and thriving is in tandem with the rise in the degree of mental health, and vice versa. This circumstance is evidently because the merger of developmental assets, creativity, and thriving plays a vital role in the promotion of healthy and positive human development. The results of this study revealed that these four factors (positive identity, support, creativity, and thriving) were important and had a significant and positive association with mental health.

Creativity comes in two forms: originality and fluency. Creativity fluency facilitates the ability of young adults to quickly generate a variety of strategies, or answers, to a specific problem, by referring to their experiences. Creative fluency also comes in handy, during the effort to decide on the most effective route, toward the solution of a problem. Among the means for enhancing creativity fluency is brainwriting (VanGundy, 1984) with post-it notes. The writing down of each idea on a separate post-it note facilitates the ensuing sharing of insights and the calculating process. As for originality, this refers to the inclination to generate perceptions that are distinctive, uncommon, and unanticipated.

Among the procedures for assessing originality is the utilization of several graders (Batey, 2012). For instance, in one exercise, specialists, with a proven track record, were called upon to assess and grade the presentations of emerging adults (Burroughs and Mick, 2004).

In spite of the adequate sample size and reliability of the scales employed, this investigation is not without several limitations. To begin with, the sample, comprising undergraduate students, cannot be taken to represent the whole undergraduate university student population of Malaysia, and neither can it represent all Malaysian emerging adults, in general, because not all Malaysian emerging adults are university students. While we consider the cooperation received for this exercise as good, this study was left wanting information, regarding undergraduate students, who declined to participate in the survey. While the impartiality of the sample cannot be verified, the cross-sectional study structure, which specifies that all measurements be performed only once, renders the establishment of causal associations beyond reach. Furthermore, the indirect associations detected during this study do not reflect statistical mediation. Furthermore, the inclusion of survey items that assessed developmental assets facilitated the examination of both internal and external assets as well as creativity and thriving to determine which factor had the strongest correlation with mental health. Although this present study used a short form of the creativity and thriving scales, extant studies indicate that the psychometric properties of the short-form scale did not differ from that of the long-form scale (Subar et al., 2001; Eisele et al., 2022).

Among the goals of this undertaking is to equip Malaysian emerging adults with the necessary tools to methodically align their creativity with contextual resources, and consequently realize healthy growth. It is our recommendation that future research, in this area, includes a systematic investigation of contextual resources, which was not covered during the course of this study. The findings delivered through this undertaking indicate that creativity can play a significant role in the development of good mental health, among individuals in emerging adulthood (Keyes, 2005). It is our recommendation that mental health professionals tasked with the development of intervention program associated with the promotion of mental health include activities that are not only stimulating but also entertaining in nature. Although this study was hampered by several limitations, distinctive contributions were delivered to existing literature regarding developmental assets, creativity, thriving, and mental health among emerging adults attending universities in Malaysia. The findings of this study have important implications for national youth policymaking and practice in Malaysia. The four factors (positive identity, support, creativity, and thriving) among university students are relevant, suggesting the crucial need for comprehensive and systematic development and implementation of national youth policy and mental health services for university students. Counselors in university environments can contribute by organizing program, specifically for the enhancement of creativity, among university students. This can facilitate their learning of new skills, for coping with problems that may crop up in their daily lives (Rahimi and Shute, 2021) and in the process thrive, as well as enhance their mental health. Mental health services and counseling should be prioritized at the institutional governance and policymaking levels and made a requirement of the university’s infrastructure. This is consistent with the idea of a “university healthy lifestyle,” where the primary concern of the institution is the health and wellbeing of its students. In terms of service delivery, universities should adopt a multi-tiered approach to promote mental health and asset-based programs to enhance creativity and thriving. Finally, institutions should provide students with organized assistance as well as opportunities to interact with their peers, feel supported by the faculty, and adjust to their new surroundings. With this present study, we confidently report that creativity and thriving are positively correlated with mental health, which is in agreement with the findings derived from an investigation conducted by Keyes (2005). The results attained through this study, can be utilized as a reference by other researchers, keen on investigating the significance of creativity and thriving among Malaysian emerging adults, and perhaps extend their investigation to cover the general population.

## Data availability statement

The raw data supporting the conclusions of this article will be made available by the authors, without undue reservation.

## Ethics statement

The studies involving human participants were reviewed and approved by the Bergen Ethics Committee. The patients/participants provided their written informed consent to participate in this study.

## Author contributions

Both authors listed have made a substantial, direct, and intellectual contribution to the work, and approved it for publication.
